# Generative AI-Powered Virtual Assistant for Guideline-Directed Medical Therapy Optimization

**DOI:** 10.1016/j.jacadv.2026.102831

**Published:** 2026-05-20

**Authors:** Syeda Beenish Bareeqa, Syed Hasham Humayun

**Affiliations:** a Department of Internal Medicine, Tidalhealth Peninsula Regional Hospital, Salisbury, Maryland, USA; b Jinnah Medical and Dental College, Karachi, Pakistan

We read with interest the paper by Navarese et al^1^ discussing the ASSIST-HF SIRIO trial, which assessed a generative AI-powered virtual assistant (VA) for guideline-directed medical therapy (GDMT) optimization in heart failure with reduced ejection fraction. While we commend the investigators for exploring innovative approaches, we have a few scientific concerns.

The scientific foundation for deploying this AI system appears inadequate. The authors mentioned a “multistage validation process based on hundreds of real-world clinical cases,” but presented no quantitative metrics regarding accuracy, sensitivity, or concordance with expert recommendations. Established frameworks like Developmental and Exploratory Clinical Investigations of DEcision support systems driven by Artificial Intelligence emphasize that early-stage clinical evaluation must include transparent reporting of AI performance metrics before human deployment.^2^ The absence of these data renders it impossible to evaluate baseline performance before patient exposure.

The VA employs retrieval-augmented generation (RAG) combined with GPT-4, yet the authors do not address known limitations of this structure. Recent systematic reviews showed that RAG systems in medical domains still result in potentially harmful answers and perform worse on case-based clinical reasoning compared to knowledge-based questions.^3^ The authors claim that RAG “reduces errors like hallucinations” but give no evidence that their implementation was tested for hallucination rates or appropriate handling of clinical edge cases.

The trial design combined multiple interventions, making it difficult to link the outcomes to the AI component specifically. The VA arm received biweekly visits compared to standard-of-care frequency, more frequent laboratory monitoring, and structured symptom evaluation. Evidence demonstrates that telemanagement encounters are independently associated with accelerated GDMT optimization, with a clear dose-response relationship.^4^ Without a control arm receiving the same visit frequency delivered by human clinicians, observed benefits cannot be attributed to the Artificial intelligence (AI) system, but rather to increased health care contact.

The authors report “no safety disagreements between VA and clinician recommendations” but provide no granular error analysis. CONSORT-AI (Consolidated Standards of Reporting Trials–Artificial Intelligence) guidelines recommend analysis of error cases, including characterization of when and why AI systems fail.^2^ The claim of zero disagreements is scientifically implausible given the complexity of GDMT titration decisions.

While AI-assisted GDMT optimization represents a promising frontier, future studies should include rigorous pre-deployment validation, appropriate control arms isolating the AI contribution, and comprehensive error analysis.

## References

[1] Navarese E.P., Leader J.H., Markides R.I.L. (2026). Design and Architecture of a Generative-AI-Supported, nonphysician-delivered model for GDMT optimization in HFrEF: the ASSIST-HF trial. JACC Adv.

[2] Rewthamrongsris P., Thongchotchat V., Burapacheep J., Trachoo V., Khurshid Z., Porntaveetus T. (2026). Evaluating retrieval-augmented generation-large language models for infective endocarditis prophylaxis: clinical accuracy and efficiency. Int Dent J.

[3] Wiest I.C., Bhat M., Clusmann J., Schneider C.V., Jiang X., Kather J.N. (2025). Large language models for clinical decision support in gastroenterology and hepatology. Nat Rev Gastroenterol Hepatol.

[4] Masanneck L., Meuth S.G., Pawlitzki M. (2025). Evaluating base and retrieval augmented LLMs with document or online support for evidence based neurology. NPJ Digit Med.

